# Supplementary material for: Are carbon emissions trading and green financial instruments synergistic? -Comprehensive quantitative research based on content analysis
Source: PLoS One. 2024 Mar 7;19(3):e0298601. doi: 10.1371/journal.pone.0298601 (PMC10919601; doi:10.1371/journal.pone.0298601)
Supplement: S1 Data — (ZIP) [file pone.0298601.s001.zip › Data/地级市Z_F工作报告.zip!/╜¡╦╒/╒≥╜¡╩╨/└┤╫╘╣1⁄2╓┌║┼í╛┬φ┐╦╩2╛▌═°í┐_2005.docx]

各位代表：

　　现在，我代表镇江市人民政府，向大会作工作报告，请予审议，并请市政协各位委员和其他列席人员提出意见。

　　2004年工作回顾

　　2004年是国家加强宏观调控、各项工作经受考验的一年。在市委的领导下，我们紧紧依靠全市人民，树立和落实科学发展观，克服困难发展，迎着挑战奋进，实现了经济社会的新进步。完成地区生产总值781.2亿元（预计数，下同），比上年增长14.7％，人均达到3535美元。财政总收入92.1亿元，增长32.6％；地方财政一般预算收入35.9亿元，增长37.5％。全社会固定资产投资320.5亿元，增长35.1％。社会消费品零售总额192.1亿元，增长14.2％。各项指标不仅完成了市五届人大二次会议确定的目标任务，而且是近五年增幅最高的一年。其中，地区生产总值、财政总收入等指标提前一年完成“十五”计划。

　　（一）为民办实事项目全面实施，完成情况总体良好

　　为民办实事项目，是政府作出的承诺，关系群众生活。面对用地控紧、银根收紧等特殊困难，经过最大程度的努力，大多数项目均超额或提前完成。我们在工作中始终做到：高度关注民生。投入2900万元充实再就业扶持基金，全年新增城镇就业2.4万人，下岗失业人员实现再就业1.4万人，其中政府购买公益性岗位720个；农村劳动力转移新增6.2万人。农村“五件实事”全面完成年度计划。农村税费改革继续深化，共调减农业税5333万元，农民人均减负30元，发放水稻直接补贴2836万元，落实良种补贴200万元；各辖市（区）均建立新型农村合作医疗保险制度，参合人口120万人，覆盖率80.1％；投资5.2亿元，建成乡村公路1080公里；改造农村草危房2763户；农村改水新增受益人口33.9万人。充分尊重民愿。沪宁铁路镇江段三线全部拆除，实现广大市民多年企盼。新增公交车71辆，新开通和优化公交线路7条。建成主城区防洪墙，整治光明河，城市防洪排涝能力得到加强。市区新增蔬菜生产基地2000亩，新建和扩建农贸市场3个。示范社区达标率和安全小区创建率分别达75％和85％，其中老住宅小区技术防范改造率达到56％，市民安全感进一步增强。努力纾解民困。实施街巷改造30条1.28万平方米、危房改造55处1.12万平方米，完成小圩子路、小米山等积水区以及黎明河二期整治。开工建设8.43万平方米经济适用房，廉租房补贴新增保障户96户，受益面扩大126％。4294名低保家庭成员免费享受医疗保险。制订并实施被征地农民基本生活保障办法，及时将农民征地补偿费清欠到位。因国家宏观调控以及城市总体规划调整等原因，市广电中心、市一中新校区、正东路健康广场等建设工程调整为跨年度工程，市体育中心立项与规划设计未能启动，目前正在全力推进。在今后为民办实事工作中，我们将更加尊重群众意愿，更加注重科学安排，一定把好事办实、实事办好，让人民群众更多地得益受惠。

　　（二）国民经济持续快速发展，综合实力明显增强

　　三次产业协调发展。一、二、三产结构调整为4.4∶58.9∶36.7。农业稳定增长。全年粮食总产量99.04万吨，增长14.9％。49个农产品获得无公害农产品认证、绿色食品标志及有机农产品认证，新增1个国家级和2个省级农业龙头企业。“三资”开发农业项目140个，总投入26亿元。土地复垦整理力度加大，新增耕地1.5万亩、复垦开发7.5万亩，实现全市耕地总量动态平衡。工业速效同增。规模以上企业完成增加值298.7亿元，增长21.9％；实现销售收入980亿元、利税76.1亿元，分别增长25.6％、23.5％。集中要素资源向规模企业、优势产业倾斜，新增产值超亿元企业37家，金东纸业、大亚集团、谏壁电厂等3家企业进入全省销售收入百强。注重培育发展后劲，完成工业性投入180.5亿元，增长50.4％，在建亿元以上项目67个，比上年增加24个。索普醋酸二期投产见效，金东3号纸机等重大项目进展顺利。服务业快速发展。完成增加值286.5亿元，增长14.2％。制订旅游业发展“三年行动计划”，全年接待境内外游客约1000万人次，旅游总收入约100亿元，分别增长46％和66％。“三山”列入国家重点风景名胜区。金融机构新增贷款余额489.3亿元，比年初增加78.4亿元。住房、汽车消费持续升温，保险、邮政通信、社区服务等行业保持较快发展。

　　对外开放克难奋进。完成合同注册外资26.8亿美元，增长49.9％；实际利用外资12.2亿美元，增长50.8％，其中已验资到位外资6.06亿美元，增长73.9％。合同注册外资和已验资到位外资增幅列苏南第一。完成进出口总额35.2亿美元，增长31.8％，其中出口15.1亿美元，增长17.1％。通过多方努力，大港三期工程即将开工建设。开发区基础设施投入18.7亿元，提高了项目的承载能力。撤销长岗收费站，出台对外商投资企业部分行政事业性收费优惠政策，投资环境进一步改善。加快实施“大通关”工程，在全国首创口岸电子政务联网，72项业务网上并联审批。完成外贸运量800万吨，集装箱16万标箱，港口货物吞吐量5302万吨。以境外工程承包为主的外经工作取得新进展。外事、侨台、经济协作等工作在扩大开放中发挥了积极作用。

　　机制活力明显增强。坚持企业发展优先、职工利益优先，完成市直国有及国有控股中小企业改制29家，改制面累计达到95.9％；落实职工经济补偿5.8亿元，调整劳动关系6.6万人。通过引进战略投资，先后实施华铝、镇纺等15个资产重组项目，焦化、江奎、船山矿等一批重点骨干企业完成转制。大港股份有限公司上市申请通过国家证监会审核。事业单位转企改制全面推进，年内已完成14家。实施市、区财税体制改革，有效防止税源流失，调动了市、区两级培植税源的积极性。民营经济蓬勃发展，吸纳民间投资161.4亿元，增长53.3％。新增私营企业2857户、个体工商户1.62万户，新增注册资本33.8亿元，民营经济增加值占地区生产总值的比重达47.6％。全面推行经营性用地市场出让制度，城市土地资产价值进一步显化，土地成交金额36.5亿元，有力支持了国企改革、城市建设以及集资兑付。

　　（三）各项社会事业协调推进，居民生活水平继续改善

　　城乡建设步伐加快。围绕“四年新提升”目标，完成南徐新城概念性规划和控制性详规，编制世业洲旅游度假区等10个专项规划，重点路段和区域控制性详规覆盖率达100％。重点工程建设进展顺利，润扬大桥南接线工程完工，沪宁高速扩建工程质量和进度全省第一，扬中长江二桥建成通车；新建和改造松村路、江滨大道、滨江旅游专线一期等城市道路，云台山等28处山体滑坡得到治理。全市已有733个行政村开通客运班车，5条县乡客运线路实现公交运营。建成天然气门站及主供气管网，2万户居民率先用上了天然气。开工建设丹徒新区污水处理厂，建成丹阳九曲河和句容赤山闸水利枢纽；丹阳西南低洼片治理工程竣工，惠及六镇80平方公里。搬迁、关闭鹤林水泥厂等4家污染企业。继续推进城管重心下移，一线保洁力量得到增强，城市保洁向郊区延伸。农村“双清双美”活动深入推进。“百日拆违”得到广大市民拥护，拆除各类违法建设13万平方米，有效遏制了违法建设蔓延势头。创建工作取得新成绩，荣获“国家环保模范城市”称号，通过“国家卫生城市”省级复核。各辖市（区）城镇建设步伐加快，城市化率达到56％，比上年提高3.7个百分点。

　　社会事业全面发展。科技进步对工业和农业的贡献率分别为48.5％和54.9％，分列全省第3位和第4位，连续第四次荣获“全国科技进步先进城市”称号。全年共实施省级以上各类科技项目210个，专利申请量超过1200件，引进大学本科以上人才2850人。国家重大科技专项“镇江城市水环境质量改善与生态修复技术研究及示范”项目推进顺利，镇江地震数字台网建成启用。制订主城区中小学布点规划，继续调整优化中小学布局。基础教育进一步巩固，高中普及率达95.1％，适龄青年中接受高等教育比例达45.3％，农村劳动力受教育年限达10.1年，在全省均居第4位。镇江博物馆新展厅建成，老馆区综合改造竣工。第一部介绍镇江人文历史的丛书——《中国历史文化名城镇江》出版发行，《镇江年鉴（2003）》获全国特等奖。承拍大型纪录片《独领风骚——诗人毛泽东》，获中国电视“星光奖”特别奖、“金鹰奖”最佳作品奖。重新编排创作音乐剧《快乐推销员》，获国家文华新剧目奖和编导奖。强化公共卫生体系和城市应急机制建设，有效防止了禽流感等重大传染病的发生。新建全民健身工程4个、全民健身点40处。成功举办第十二届市运会、第二届残运会，承办全国女排大奖赛等重大赛事。开展城市精神大讨论，确立“创业创新、开放文明、务实诚信”的新时期镇江精神。深化双拥和国防教育，军政、军民关系更加融洽。统计、新闻出版、档案等工作取得新进步，老龄、妇女儿童、关心下一代等事业实现新发展。

　　富民惠民成效显著。把富民作为政府工作的重中之重，全力落实各项富民政策措施。城镇居民人均可支配收入突破万元大关，达到10858元，增长14.9％；农民人均纯收入5372元，增长突破两位数，达到13.5％。城乡居民收入增幅均?断完善，城镇养老、失业、医疗三大保险参保率均提高到98％，居全省前列，养老保险首次实现年度收支平衡。城市低保实现“应保尽保”，标准统一提高到每人每月200元。农村最低生活保障覆盖面扩大，4725户困难农民家庭受益。大力开展医疗救助，设立惠民医院，对低收入居民就医实行优惠。

　　（四）民主法制建设继续推进，政府自身建设得到加强

　　按照“创新、服务、高效、规范、清廉”的总体要求，努力建设人民满意的政府。积极贯彻实施《行政许可法》，建立市政府组成人员两月一期集中学法制度。深化行政审批制度改革，再次取消各类审批事项78项，改变管理方式40项。市行政服务中心一年来共受理申报事项90765件，办结率达99.9％，群众满意率98.7％。为民服务公开电话接市民投诉5415件次，办理5145件，办结率95.1％；处理政府网上信箱信件3225件。政务公开继续在全国保持领先水平，机关事务管理工作受到国务院表彰。积极推进“平安镇江”创建工作，深入开展严打整治斗争和社会治安综合治理，社会治安总体水平位居全省前列，全市社会安定、投资安全、人民安宁。安全生产形势平稳，死亡事故和死亡人数实现“双下降”。高度重视群众来信来访和人民调解工作，努力处理好各类群体事件和信访问题。认真落实市人大及其常委会的决议决定，自觉接受市人大及其常委会的法律监督和工作监督、市政协的民主监督以及社会监督，办理人大代表建议186件、政协提案419件。

　　过去的一年，我们坚持用科学发展观审视和指导工作实践，积极应对宏观环境变化。在目标定位上，坚持率先发展、加快发展不动摇。自觉拉高标杆，层层传递压力，在现实的困难面前、在发展的紧要关头，始终保持奋发作为的精神状态，做到速度能快则快、目标能超则超。在发展思路上，坚持在科学发展的前提下率先发展。更加注重以人为本，认真落实中央和我市两个“1号文件”，把富民放在首位，全年社保、优抚等公共财政支出11.5亿元，增幅超过60％。更加注重结构调整，加快转变经济增长方式，优化开发区功能布局，坚持引资与引技并重，高新技术产业化与传统产业高新化并重，高新技术产业增加值占全市规模以上工业增加值的比重达到28.8％，比上年提高2.２个百分点。更加注重城乡统筹，把“三农”工作放在全局工作的突出位置，积极探索以城带乡、以工促农的发展机制，全年财政支农3.75亿元，增长14％。在工作推进上，敢于直面矛盾，创新发展思路，积极破解难题。用地方面，不等增量用活存量，盘活存量土地8500多亩，开发区兴建多层标准厂房13万平方米，提高集约利用水平；用电方面，立足自身精细调度，鼓励地方电厂多发满发，同时积极向上争取指标，提高电力配置效率。通过充分发挥主观能动性，在被动中争主动，在应变中求发展。

　　回顾过去一年，成绩确实来之不易。我们真切地感到，政府工作每向前推进一步，各项事业每取得一点成绩，都凝聚着全市人民的智慧和力量，都倾注了全体劳动者的心血和汗水。在此，我代表市人民政府，向全市人民，向各位人大代表、政协委员，向市各民主党派、工商联、人民团体、各界人士、离退休老同志，向驻镇解放军、武警官兵和公安干警，向垂直部门和驻镇单位，向在镇的外来建设者和所有关心、支持镇江发展的海内外朋友，表示衷心的感谢并致以崇高的敬意！

　　各位代表，在总结成绩的同时，我们也清醒地看到，我市经济社会发展中还存在不少薄弱环节，面临的矛盾和困难还很多。突出表现在：城乡居民富裕程度还不高，全民创业氛围不浓，农民增收的长效机制尚未形成；经济结构性矛盾仍然比较突出，区域经济竞争力还不够强；城市的历史、文化、商业等优势资源还没有得到充分的挖掘与整合，城市特色和城市品牌未能充分彰显；居民拆迁和被征地农民养老、转岗就业矛盾还很突出，影响社会稳定的因素依然存在；政府职能转变还不能完全适应市场经济的要求，行政效率、服务水平有待进一步提高；等等。我们将正视这些薄弱环节和矛盾问题，增强忧患意识和紧迫感，开拓创新，积极进取，在推进改革和发展中努力加以解决。

　　2005年工作任务

　　2005年是全面实现“十五”计划、衔接“十一五”发展的关键一年。做好今年工作，意义重大、事关长远。我们将以邓小平理论和“三个代表”重要思想为指导，深入贯彻党的十六届四中全会和中央经济工作会议精神，按照市委四届十次全会提出的“全面提升、全力争先”的总体要求，以科学发展观统揽全局，更加注重率先发展、富民惠民，更加注重统筹、协调、可持续发展，进一步发挥沿江优势，加快结构调整，打造生态城市，构建和谐社会，推动镇江经济社会更快更好发展。

　　全市国民经济和社会发展的预期调控目标为：地区生产总值增长13％；全社会固定资产投资增长20％；财政总收入增长15％，地方财政一般预算收入增长15％；合同注册外资和实际到位外资分别增长20％和25％；进出口总额增长15％，其中出口增长13％；社会消费品零售总额增长13％；城镇居民人均可支配收入增长12％，农民人均纯收入增长9％；城镇登记失业率控制在4％以内；人口自然增长率控制在2‰以内。工作中我们将力求做得更好。

　　同时，着力办好15件实事：

　　1.新增城镇就业2.5万人，农村劳动力转移6万人，进一步推进富民工程；

　　2.全面完成农村“五件实事”，促进城乡共同发展；

　　3.全面实施农村居民最低生活保障制度，做到“应保尽保”；

　　4.整治主城区积水区，解决张王庙沟、江滨村村民九组、车站西一村、绿竹巷等片区汛期内涝问题；

　　5.改造市区10个老住宅片区、80条街巷道路，进一步改善人居环境；

　　6.完成市区4处山体滑坡治理工程，防止城市斜坡地质灾害事故；

　　7.加快天然气工程建设，完成天然气转换6万户；

　　8.加快治安防控体系建设，全市老住宅小区技术防范设施改造达80％，农村契约化保险式联防达90％，所有学校设立警务站（室）；

　　9.实施食品安全工程，建设“放心粮店”40家，在市区58家连锁超市实施“放心菜、放心肉”工程，进一步提高居民食品供应质量；

　　10.市区新增60辆公交车，新辟和调整优化6－8条公交线路，新建和改造公交站棚30－35座；

　　11.开展艾滋病、结核病、血吸虫病“三病”防治工作，提高人民群众健康水平；

　　12.完成市儿童医院一期工程，改善儿童就医条件；

　　13.新建市实验初级中学，解决黄山南路周边地区“入学难”问题；

　　14.实施钛白粉厂、上铁水泥厂等污染企业搬迁，改善环境质量；

　　15.开通“中国镇江”门户网站，为市民和外商提供网上公共服务。

　　围绕上述目标，我们将扎实做好以下六个方面的工作：

　　（一）全力实施富民工程，增加城乡居民收入

　　抓好就业再就业工作。把就业作为经济社会发展的优先目标，实行更加积极的就业政策，营造更加宽松的创业环境。注重发展劳动密集型企业，积极开发公益性岗位，创造更多的就业机会。加大再就业资金筹措力度，继续推进政府差额购买就业岗位，帮助特殊困难群体就业再就业。进一步落实税费减免、小额贷款、社保补贴等再就业扶持政策，鼓励企业吸纳下岗失业人员，鼓励下岗失业人员自谋职业、自主创业。规范城乡劳动力市场，推广各类职业技术培训，推进城乡统筹就业。

　　提高居民收入水平。形成稳定的收入增长机制，健全最低工资保障制度，推行工资集体协商制度，增加从业人员的工资性收入。引导和鼓励居民将储蓄转化为有效投资，提高经营性、资产性收入在居民收入中的比重，扩大中等收入群体。进一步突出非农产业在农民增收中的主体作用，用好1000万元农民创业担保基金，推进农村劳动力向非农产业转移，多渠道增加农民收入。

　　完善社会保障体系。加大城镇企业养老保险扩面力度，出台乡镇企业参加城镇养老保险办法，全年养老保险扩面2万人，养老保险基金征缴率95％以上，努力实现应保对象保障率、保障经费到位率和保障待遇兑付率“三个100％”。完善被征地农民基本生活保障制度，建立农村医疗救助制度。优化房地产开发结构，增加面向中低收入家庭的商品住房供应；完成1万平方米危房治理，继续做好特困家庭的住房保障。在全社会积极开展扶贫济困活动，成立镇江慈善总会，让更多有社会责任心、有爱心的人参与公益事业，让更多应该受到关爱的人得到关爱。

　　（二）加快推进结构调整，增强产业竞争能力

　　提高农业发展水平。实行最严格的耕地保护制度，加大以防汛抗旱为重点的农业基础设施投入，加快农业技术推广，改善农业装备水平，保护和提高农业综合生产能力。全面免征农业税，继续落实粮食直补、良种直补、农机补贴等各项政策措施，充分调动农民种粮积极性。积极引导“三资”开发农业，力争全年投入30亿元。培育壮大农业龙头企业，帮助企业与农民建立稳定密切的利益联结机制，促进农业向规模化、品牌化、产业化方向发展。进一步?经济。坚持走新型工业化道路，加快信息化带动工业化步伐，推动产业优化升级，提高工业经济整体素质。突出有效投入，完成工业性投入230亿元，增长28％以上，占全社会固定资产投资的比重达到60％。重点培育50强企业，力争销售超50亿元企业达到4家，确保规模以上企业完成增加值超350亿元、增长20％以上，形成一批新增长点。突出培育产业集群，以重点骨干企业为龙头，加强产业规划引导，在造纸、化工、五金工具、汽摩配件、工程电器等方面，形成一批具有区域品牌效应的特色产业群。突出科技的先导带动作用，加快高新技术产业“一园四基地”和省级高新技术产业园区建设，继续实施“百企创新工程”。力争高新技术产品销售收入增长25％以上，新增省级以上高新技术企业20家，企业专利申请数增长15％，增强企业的核心竞争力。

　　提升服务业发展层次。积极实施旅游业“三年行动计划”，着力建设“三山”国家重点风景名胜区、南山生态休闲区、世业洲旅游度假区和西津渡历史街区四大板块。抓紧北固山景区建设前期准备，做好茅山风景区申报国家重点风景名胜区工作。以润扬大桥建成通车为契机，积极发展第三方物流、连锁经营等新型商贸业态，引进沃尔玛等大型超市落户镇江，提高宾馆、餐饮等服务行业的接待能力和水准。建设市农副产品批发市场、润扬国际商贸城等重点工程，推进第一楼街“商业步行街”、大西路“传统商业一条街”等特色街区建设。优化信贷结构，建立多层次的信用担保体系，积极引进市外金融机构，大力发展保险、证券业务，利用资本市场扩大直接融资比重。

　　（三）积极实施沿江开发，提高对外开放水平

　　按照“区域集中、产业集聚、开发集约”的原则，紧紧围绕产业、园区、基础设施三个关键环节，突出招商引资，狠抓重点项目，使沿江地区成为对外开放的重要载体和经济增长极，以大开发促进大开放。

　　推进开发区建设。把开发区建设作为政府的一项重要工作。进一步细化分区规划和配套专项规划，强化产业特色，吸引项目进区，确保开发区利用外资占全市总量的50％以上。充分发挥出口加工区的优势，吸引加工贸易企业落户。加强坍江整治，加大对长江岸线的清理、整合力度，制订岸线集约利用的具体办法；加大基础设施投入，加快大港三期等5个万吨级码头建设和谏壁电厂、镇江电厂等能源项目建设，提升产业集聚的配套服务功能。

　　提高招商成效。调整招商策略，突出对台港地区的招商工作，加大对日韩的引资力度，积极吸引欧美资本，主攻大公司、大项目和高新技术项目，力争引进总投资超亿美元项目8个。围绕机电、化工、造纸、新材料等优势产业，进一步整合招商资源，加大产业链招商力度。完善服务体系，优化投资环境，促进现有企业增资扩股和“以外引外”。在抓好工业项目招商的同时，重视农业、服务业和社会事业领域的招商工作。加大领导力度，明确引资责任，强化工作考核。积极探索市场化运作的招商机制，加强专业招商队伍建设，落实招商引资激励政策，提高项目引进成功率。

　　拓展外贸外经。完善外贸出口促进体系，提高出口产品的档次和附加值，鼓励采用本地货源的一般贸易出口，推动出口快速增长。实施“走出去”战略，帮助更多的企业获得对外承包劳务经营权，壮大外经合作主体。支持有条件的企业对外投资和跨国经营，带动产品出口和劳务输出。

　　（四）不断深化城乡改革，激发体制机制活力

　　巩固企业改革成果。按照改制规范要求，督促改制企业完善后续工作，维护好职工的合法权益。妥善处理改制企业的遗留问题，加快破产、关闭企业资产处置和人员分流。引导企业完善法人治理结构，深化内部配套改革，建立现代企业制度。坚持阳光操作，通过增量式、开放式改革，促进企业扩张规模、提升质量。推动企业通过多种形式直接融资，力争实现1－2家企业上市。

　　放手发展民营经济。突出本土创业和引进民资两大工作重点，更好地发挥政府扶持、协调、服务功能。加快建立民营经济统计体系，完善投诉、督查和服务工作机制，进一步做好融资担保、技术支持和权益保障等工作。实现全市担保基金和担保业务规模“翻一番”。力争新增私营企业3360户、个体工商户1.92万户，吸纳民间投资200亿元，增长25％，民营经济增加值占地区生产总值的比重提高5个百分点以上。

　　协调推进各项改革。理顺国有资产监督管理体制，落实国有资产经营责任，确保国有资产保值增值。加快投资体制改革，全面确立企业投资主体地位，合理界定政府投资范围，加强对全社会投资的引导和调控。按照“分类定位、分开管理、分别改革”的思路，加快事业单位改革，年内完成生产经营类事业单位转企改制任务。积极稳妥地推进自来水、燃气、污水处理、公交等市政公用事业改革。深化农村税费改革、乡镇财政管理体制改革和水利体制改革。

　　（五）突出山水生态建设，提升城市对外形象

　　围绕建设“山水镇江、生态镇江、园林镇江、人文镇江”的总目标，以“启动新城、改造老城、彰显特色、完善功能”为主题，进一步充实城市框架，注重景观建设，提升建筑品位，精心打造山水生态园林城市。

　　拓展城市空间。启动南徐新城建设，新建九华山路、团山路“一纵一横”两条骨干道路，着手进行有关场馆的选址设计。建设经十二路南延工程，完善城东发展轴。呼应润扬大桥通车，上半年完成跃进路工程，新建戴家门路，形成城市西外环。拓宽改造黄山北路，实现南山“北水”一路贯通。实施滨水内环线西段、石马路、镇荣公路北延、万古一人路、中山北路等工程，进一步改善城市道路系统。拓展城市对外快速通道，加快实施沪宁高速扩建、镇溧高速新建、312国道改建等工程，积极推进镇泰过江通道、禄口机场通道及城际轨道交通建设，加快融入长三角的步伐。加大市场化运作力度，在做好经营土地文章的同时，激活变现城市资产，拓展城建投资渠道。

　　加强生态建设。积极发展循环经济，推行清洁生产，开展生态示范区建设，全面启动生态市创建工作。强化沿江、沿河、沿山、沿路绿地和林带建设，建设滨江旅游风光带，续建跃进河、团结河风光带。新增城市绿地100公顷，建成区绿化覆盖率达到37.2％，创建“国家园林城市”。加大“水环境专项”实施力度，保护好各类湿地和水体。加强山体资源保护，严禁非法开山取石，做好采石宕口综合整治。加快各辖市创建环保模范城市步伐，努力建设环保模范城市群。

　　提高城管水平。完善城市管理重心下移体制，健全综合执法、长效管理机制。实行市政、绿化“管办分离”，推行城市道路养护、绿化养护和环卫保洁市场化运作。继续抓好违法建设整治，开展占道经营、“三小车”等专项整治。优先发展城市公共交通，开通镇扬城际公交，优化公交运输网络。加强市区停车设施的规划建设，逐步解决市民停车难的问题。

　　（六）统筹经济社会发展，全力打造和谐社会

　　加强精神文明建设。以争创全国文明城市为龙头，统筹各项创建活动，深入推进“现代文明市民教育工程”，突出抓好以诚信为重点的公民道德建设，提高市民文明素质；加强和改进未成年人思想道德建设，指导和促进未成年人健康成长；充分发挥社区了解人、关心人、帮助人、凝聚人的作用，使社会更温馨、更和谐。

　　坚持科教兴市。围绕构建区域技术创新体系，抓好生产力促进中心、高新技术创业中心和工程技术研究中心三大平台建设，积极申报实施一批国家级和省级技术创新项目。加快人才和人力资源开发，不断完善和创新人才政策，开辟引进各类高层次和紧缺专业人才“绿色通道”。坚持素质教育方向，加强教师队伍建设，努力提高教育质量。进一步调整教育布局、改善办学条件，加快一中新校区建设，启动十一中新校区工程。完善以辖市（区）为主的农村义务教育管理体制，推动城乡教育和区域教育优质均衡发展。积极支持在镇高校的建设与发展。加强职业技术教育和培训，大力发展各类继续教育，努力构筑现代终身教育体系，打造学习型社会。

　　协调发展社会事业。强化政府公共卫生管理职能，增加公共卫生投入。按照便民利民的原则，加快城镇医药卫生体制改革，扩大农村新型合作医疗受益面，年内基本建成覆盖城乡、功能完善的疾病预防控制和医疗救治体系。深入开展计划生育优质服务，加强流动人口计划生育管理。按照“突出重点、彰显个性、做出亮点”的要求，挖掘整理具有镇江个性特点的历史文化，展示名城的人文魅力。建成苏南抗战胜利纪念碑二期工程、茅以升纪念馆。开工建设电台发射台，完成市体育中心建设规划、立项等前期准备工作。努力办好十运会部分承办项目，积极备战十六届省运会，提高竞技体育水平。开展“场地建在百姓身边、体育走进千家万户”活动，组织实施全市国民体质监测工作，建成全民健身工程（点）30个。新建市气象雷达站，提高突发性、灾害性气候预报的时效和准确率。深入开展全国民族团结进步模范城市创建工作，争创全国双拥模范城“五连冠”。

　　推进民主法制建设。坚持向市人大及其常委会报告工作、向市政协通报情况，自觉接受监督。加强与各民主党派、工商联、无党派人士及各人民团体的联系，广泛??，完善村民居民自治和企事业单位民主管理制度。全面实施“法治镇江”建设规划，强化社会治安综合治理，建成全省首批“平安市”。依法严厉打击制售假冒伪劣商品等违法犯罪行为，维护市场经济秩序。严格落实安全生产责任制，努力减少各类事故发生。完善应急救援和指挥系统，增强对突发事件和各种灾害的处理能力。依法维护劳动者的合法权益，加强对外来务工人员的管理和服务，营造和谐的劳动关系。进一步加强信访工作，完善“大调解”机制，妥善处理涉及群众利益的各类矛盾。

　　加强政府自身建设

　　围绕“创新、服务、高效、规范、清廉”十字目标，进一步转变政府职能，提高行政效能，改进工作作风，致力建设忧民所忧、乐民所乐的民本政府，务实高效、勤政廉洁的责任政府，依法行政、公正严明的法治政府。重点在以下几个方面提升水平：

　　进一步增强执政为民意识。执政为民，字字千钧。市政府全体组成人员和公务员，都要自觉顺应发展变化的形势，按照人民的期盼，全身心地投入工作。带着责任抓学习，不断更新知识结构，提升思维层次，提高处理各种复杂问题的能力。带着使命促发展，本领用在干事业上，功夫下在抓落实上，把镇江的事情办好，对镇江人民负责。带着感情帮民富，与群众想在一起、干在一起，确保人民生活水平每年都有新提高。

　　进一步推进科学民主决策。完善公众参与、专家论证和政府决策相结合的决策机制。对“十一五”规划编制等涉及经济社会发展全局的重大事项，深入调查研究，广泛征询意见，做到问政于民；对与群众日常生活和切身利益密切相关的社会事务，实行公示、听证等制度，并通过市长信箱、政府公开电话等途径，促进政府与市民的互动交流，做到听政于民；及时向社会公开政府履行职责情况，继续办好“行风热线”，接受纳税人和群众的评议与监督，做到议政于民。以公开民主，促科学决策，使政府工作更加切合实际，更加顺民意、得民心。

　　进一步强化规范高效服务。认真贯彻国务院《全面推进依法行政实施纲要》，理顺行政执法体制，建立健全各项制度，依照法定权限和程序履行职责、行使权力，着力解决职能交叉、多头执法、重复处罚等问题。完善市行政服务中心运行机制，加快辖市（区）、乡镇（街道）服务中心建设，形成三级便民服务网络，缩短行政审批流程，提高机关办事效率，降低社会各界到政府部门办事的制度性成本。加快电子政务建设，统一构建政府网络平台，开通市到各辖市（区）的电子政务纵向网，实现市与辖市（区）及市级机关之间公文无纸化传输，丰富交互式信息和网上受理内容，为企业、投资者和市民提供便捷的多功能服务。

　　进一步塑造清正廉洁形象。坚持公正公道、正派正义，掌权为公、用权为民。认真落实党风廉政建设责任制、述职述廉、罚缴分离、政府采购等各项制度，充分发挥监察、审计、财政等监管职能，对政府投资项目实行预算审查、公开招标和竣工审计，使政府权力在制度轨道中规范运行。珍惜民资民力，坚持勤俭办事，压缩一般性支出，把有限的资金用在最急需发展的事业上。深入开展反腐败斗争，坚决查处违纪违法案件，提高政府公信力。

　　各位代表，镇江正进入一个新的重要发展时期，时代的重任召唤我们奋发进取，人民的期望不容我们丝毫懈怠。让我们高举邓小平理论伟大旗帜，全面贯彻“三个代表”重要思想，在中共镇江市委的坚强领导下，团结和依靠全市人民，励精图治，扎实工作，为全面完成今年的各项目标任务而奋斗，把我们共同热爱的镇江建设得更加美好！
